# Pneumococcal Meningitis Complicated by Spinal Cord Dysfunction and Acute Polyradiculopathy

**DOI:** 10.31486/toj.18.0153

**Published:** 2020

**Authors:** Mohamed A. Abdallah, Khalid Mohamed Ahmed, Maria Victoria Recio-Restrepo, Mowyad Khalid, Ahmed Yeddi, Ahmad Abu-Heija, Mazin Khalid

**Affiliations:** ^1^ Department of Medicine, University of South Dakota Sanford School of Medicine, Sioux Falls, SD; ^2^ Department of Internal Medicine, Wayne State University/Detroit Medical Center, Detroit, MI; ^3^ Department of Medicine, Interfaith Medical Center, Brooklyn, NY

**Keywords:** *Meningitis–pneumococcal*, *neurologic manifestations*, *paraplegia*, *polyradiculopathy*, *Streptococcus pneumoniae*

## Abstract

**Background:** Meningitis caused by *Streptococcus pneumoniae* is associated with devastating clinical outcomes. A considerable number of patients will develop long-term neurologic complications. Hearing loss, diffuse brain edema, and hydrocephalus are frequently encountered. Acute spinal cord dysfunction and polyradiculopathy can develop in some patients.

**Case Report:** A 63-year-old female was admitted to our hospital with sudden-onset bilateral lower extremity weakness. On admission, the patient had evidence of spinal cord dysfunction given the abnormal motor and sensory physical examination findings and the absent sensation with a sensory level at dermatome T4 on neurologic examination. Computed tomography myelography did not show evidence of spinal cord compression or transverse myelitis. Cerebrospinal fluid examination was positive for pneumococcal meningitis. The patient was treated with antibiotics and steroids. Nerve conduction studies demonstrated the absence of response, suggesting damage to the peripheral nerves and polyradiculopathy. The patient was treated with plasmapheresis for possible Guillain-Barré syndrome; however, she did not improve despite appropriate antibiotics, steroids, and plasmapheresis. She developed persistent quadriparesis, sensory impairments in upper and lower extremities, and bowel and bladder sphincter dysfunction.

**Conclusion:** Our case demonstrates the development of spinal cord dysfunction (supported by the sudden onset of paraplegia and the presence of a sensory level) and polyradiculopathy (flaccid paralysis, ascending weakness, and absence of response in neurophysiologic studies suggesting severe damage to the peripheral nerves). The appearance of either complication is unusual, and the simultaneous occurrence of both complications is even more uncommon.

## INTRODUCTION

The in-hospital mortality of patients with meningitis caused by *Streptococcus pneumoniae* is estimated to be approximately 25%, and fewer than 50% of patients will have a good outcome on hospital discharge. Intracranial and neurologic complications are common, with rates reported as high as 75% in some case series.^1^

Acute spinal cord dysfunction caused by myelitis and polyradiculopathy are rare neurologic complications that can develop in patients with bacterial meningitis. The simultaneous occurrence of both complications following bacterial meningitis is even more unusual, with only a few reported cases.^1^ We present a case of acute lower extremity weakness with features of spinal cord dysfunction and polyradiculopathy in a patient with pneumococcal meningitis.

## CASE REPORT

A 63-year-old female presented to the emergency department (ED) with bilateral lower extremity weakness. Muscle weakness was sudden, involved both limbs at the same time, and did not involve other muscle groups. She also reported fever, body pains, and feeling unwell for approximately 4 days prior to admission but denied any recent history of diarrhea. Evaluation in the ED showed bilateral lower extremity weakness with inability to perform any voluntary movements and bladder dysfunction with the inability to urinate. The patient had multiple chronic medical problems, including type 2 diabetes, hypertension, and morbid obesity with a body mass index of 68 kg/m^2^. She had been recently diagnosed with chronic lymphocytic leukemia but was not on treatment.

Physical examination confirmed absent sensation with a sensory level at T4, paraplegia with a power of 0/5 bilaterally, absent deep tendon reflexes, and a negative Babinski sign. Because of the patient's weight, magnetic resonance imaging (MRI) of the spine could not be performed. Computed tomography (CT) myelogram (cervical, thoracic, and abdominal spine) was negative for cord compression and transverse myelitis. An indwelling urethral catheter was inserted for urine retention.

Three days after admission to the hospital, the patient reported worsening of symptoms, principally muscle weakness and sensory disturbances with a burning sensation at the abdomen that extended to the lower extremities. She developed respiratory failure requiring intubation and mechanical ventilation. The patient also developed a fever of 38.1°C at that time. Blood and urine cultures were sent, and the patient was started empirically on intravenous (IV) vancomycin and meropenem, both dosed at 2 g every 8 hours; the vancomycin trough levels goal was 15 to 20 mcg/mL. IV methylprednisolone was also started for suspected transverse myelitis, although a diagnosis of Guillain-Barré syndrome (GBS) was not excluded at that time. The patient's complete blood counts and chemistries were unremarkable. Cerebrospinal fluid (CSF) was significant for white cells of 94 cells/mm^3^ (reference range, 0-3 cells/mm^3^), protein of 677 mg/dL (reference range, 50-80 mg/dL), and glucose of 152 mg/dL (reference range, 15-45 mg/dL) (Table). Both urine and blood cultures grew *Streptococcus pneumoniae*, and CSF polymerase chain reaction test was positive for *Streptococcus pneumoniae*.

**Table.** Cerebrospinal Fluid Analysis Results

Parameter	Result	Reference Range
Pressure, cmH_2_O	Normal	5-20
Appearance	Turbid	Clear
White blood cells, cells/mm^3^	94 (75% lymphocytes)	0-3
Protein, mg/dL	677	50-80
Glucose, mg/dL	152 (57% of serum glucose)	15-45 (>50%-75% of serum glucose)
Gram stain	Negative	Negative
Cultures	Negative	Negative

Pneumococcal meningitis was diagnosed; however, the clinical picture was suggestive of GBS. Steroids were stopped after 3 days, and the patient was started on plasmapheresis for suspected GBS. Nerve conduction studies of the lower extremities showed an absence of response, suggesting damage to the peripheral nerves and polyradiculopathy. Vancomycin and meropenem were switched after 4 days of initiation to IV ceftriaxone 2 g twice daily.

The patient was on mechanical ventilation for a total of 25 days. Because she could not be weaned off mechanical ventilation, the patient required a tracheostomy and percutaneous endoscopic gastrostomy tube insertion 1 week after intubation. She completed 10 sessions of plasmapheresis with no neurologic improvement. During the following weeks of hospitalization, the patient had intermittent episodes of fever and 3 episodes of bilateral lower extremity cellulitis that were treated with vancomycin 2 g every 8 hours and cefazolin 1 g every 8 hours. Because of the persistence of fevers while the patient was receiving antibiotics for cellulitis, blood cultures and imaging were repeated (including CT of the head); however, no additional source of infection was revealed. Lumbar puncture was repeated 18 days after the initial results, and CSF analysis showed a protein count of 67 mg/dL (reference range, 50-80 mg/dL) and a cell count of 25 cells/mm^3^ with 87% lymphocytes (reference range, 0-3 cells/mm^3^), suggesting no evidence of new meningitis. The fever was thought to be secondary to vancomycin administration (drug fever).

The patient was successfully weaned off the ventilator and was transferred to an inpatient rehabilitation facility after spending 32 days in the hospital. The patient sustained bilateral upper and lower extremity weakness, sensory loss below T4 that persisted for at least 2 months from hospital discharge, and constipation and chronic urinary retention. The patient was discharged from the inpatient rehabilitation facility to a long-term acute care facility. Six months after hospital discharge, motor function of her upper/lower extremities had not improved, and the patient could not perform purposeful movements using upper or lower extremities. The patient had a modest improvement in respiratory muscle function and return of minimal sensations in the upper extremities. She continued to have sensory impairment in the lower extremities, in addition to persistence of bowel and bladder sphincter dysfunction with constipation and urine retention that required placement of an indwelling urethral catheter. After the 6-month follow-up, the patient was lost to follow-up when she moved out of state.

## DISCUSSION

Bacterial meningitis is known to cause focal neurologic deficits. Spinal cord involvement, however, remains rare with an estimated incidence of approximately 2% in some case series.^1^ Complications include brain edema,^1^ hydrocephalus,^1^ hearing loss,^1^ intramedullary spinal cord abscess,^2,3^ epidural abscess compressing the spinal cord,^4^ spinal cord infarction,^5^ direct spread of the infection,^6^ nonspecific myelitis,^7^ and vasculitis.^8^

Bacterial meningitis complicated by isolated polyradiculopathy has also been reported in the literature. In one case report, a 17-year-old male admitted with meningococcal meningitis developed paraplegia, and his spine MRI and nerve conduction studies were consistent with acute inflammatory demyelinating polyneuropathy (AIDP).^9^ He completely recovered with no specific treatment given other than antibiotics for the meningitis. In another case report, a 14-month-old female with pneumococcal meningitis showed lumbosacral polyradiculopathy on enhanced spine MRI that manifested clinically as paraplegia and bladder/bowel dysfunction.^10^ The case of a 68-year-old female with invasive pneumococcal disease (meningitis, endocarditis, and pneumonia) was complicated by quadriparesis; nerve conduction studies showed axonal damage involving both motor and sensory nerves in all 4 extremities.^11^ The patient regained normal function in her upper extremities within 6 months of initial insult, but flaccid paralysis of both lower extremities persisted. She did not regain bowel or bladder sphincter function.^11^

Involvement of both the spinal cord and nerve roots/peripheral nerves simultaneously as a complication of tuberculous (TB) meningitis has been reported.^12^ This complication remains very rare in acute bacterial meningitis. In one case, a 35-month-old female with *Listeria* meningitis had neurophysiologic studies consistent with AIDP, and her spine MRI showed grey matter signal enhancement in the entire spinal cord.^13^

Our case has features of spinal cord dysfunction (supported by the sudden onset of paraplegia and the presence of a sensory level) and features of polyradiculopathy (flaccid paralysis, ascending weakness, and absence of response in neurophysiologic studies suggesting severe damage to the peripheral nerves). In a study of myeloradiculopathy complicating TB meningitis, elevated CSF protein was an important predictor of spinal nerve root involvement.^14^ In that study, involvement of the spinal cord and spinal nerve root in TB meningitis was associated with a poor prognosis for recovery.^14^

## CONCLUSION

Spinal cord dysfunction and polyradiculopathy are rare but severe complications of pneumococcal meningitis. Physicians should be aware of these complications because they are associated with a poor prognosis.

## Notes

This article meets the Accreditation Council for Graduate Medical Education and the American Board of Medical Specialties Maintenance of Certification competencies for Patient Care and Medical Knowledge.
